# Quantification of relative neurite tortuosity using Fourier transforms

**DOI:** 10.1016/j.jneumeth.2021.109266

**Published:** 2021-06-22

**Authors:** Benjamin Smith, Ananya Datta, Justin Lee, David Evans, Suzanne Fleiszig

**Affiliations:** a School of Optometry, University of California, Berkeley, CA 94720, USA; b Graduate Program in Vision Science, University of California, Berkeley, CA 94720, USA; c College of Pharmacy, Touro University California, Vallejo, CA 94592, USA

**Keywords:** Neurite, Tortuosity, Segmentation, Fourier transform, Fluorescence microscopy, Cornea

## Abstract

**Background:**

The tortuosity of nerve fibers has been shown to be important for identifying and monitoring clinically relevant manifestations resulting from of a variety of ocular and systemic disease pathologies and disorders. However, quantifying tortuosity in dense neurite networks can prove challenging, as existing methods require manual scoring and/or complete segmentation of the neurite network.

**New method:**

We measured neurite tortuosity by quantifying the degree of directional coherence in the Fourier transform of segmented neurite masks. This allowed for the analysis of neurite tortuosity without requiring complete segmentation of the neurite network. We were also able to adapt this method to measure tortuosity at different length and size scales.

**Results:**

With this novel method, neurite tortuosity was accurately quantified in simulated data sets at multiple length scales and scale variant and scale invariant tortuosity was accurately distinguished. Use of this method on images of murine corneal neurites correctly distinguished known differences between neurite tortuosity in the peripheral and central cornea.

**Comparison with existing method(s):**

Other methods require complete segmentation of neurites, which can be prohibitive in dense and/or sparsely labeled neurite networks such as in the cornea. Additionally, other methods require manual curation, manual scoring, or generation of a curated training set, while our novel method directly measures tortuosity as an intrinsic property of the image.

**Conclusions:**

We report the use of Fourier transforms for quantification of neurite tortuosity at multiple length scales, and with an image input that contains incompletely segmented neurites. This new method does not require manual training or curation, allowing a direct and rapid measurement of neurite tortuosity, thereby enhancing the accuracy and utility of neurite tortuosity measurements for evaluation of ocular and systemic disease pathology.

## 1. Introduction

### 1.1. Clinical relevance of neurite tortuosity

*In vivo* confocal microscopy (IVCM) serves as a noninvasive imaging tool, allowing for both structural and quantitative analyses of the cornea (Jalbert et al., 2003). In particular, images of the sub-basal nerve plexus, the primary nerve plexus supplying the corneal epithelium, collected by IVCM have been utilized to evaluate various aspects of corneal nerve morphology. The tortuosity of these nerve fibers has been shown to be an important indicator of clinically relevant changes resulting from a variety of disease pathologies and disorders. For example, corneal nerve tortuosity was cited as a key biomarker for assessing the severity of diabetic neuropathy (Edwards et al., 2014). Nerve tortuosity was inversely correlated with corneal sensation, a primary symptom of Herpes zoster ophthalmicus infection of the trigeminal nerve (Hamrah et al., 2013). Patients with acute *Acanthamoeba* keratitis or fungal keratitis demonstrate a significant increase in nerve tortuosity (Kurbanyan et al., 2012), and abnormally tortuous nerve fiber bundles were found in patients with primary Sjögren’s syndrome (Tuominen et al., 2003).

### 1.2. Limitations of previous methods

Previous studies have used two primary methods for evaluation of nerve tortuosity. One method that bypasses the need for image processing involves assessment by a corneal specialist or trained grader who assigns images a grade of nerve tortuosity, or a normal/aberrant status (Hamrah et al., 2013; Muraoka et al., 2013; Eleid et al., 2014; Lagali et al., 2015). However, tortuosity estimations produced by specialists or graders are limited by inter-observer and intra-observer variability, as well as limited repeatability due to cost and time. Alternatively, automated methods have been presented, and in recent years have taken precedence over manual segmentation. The most common method of automatic tortuosity estimation framework (ATEF) involves the use of a variety of different algorithms (Guimarães et al., 2014, 2016; Joshi et al., 2010; Wilson et al., 2008; Bullitt et al., 2004; Koprowski et al., 2012; Scarpa et al., 2011; Grisan et al., 2008; Annunziata et al., 2016).

A fundamental issue with tortuosity estimation through an ATEF is their dependence on complete, correct segmentation of all neurites within an image, making them unamenable to dense or complex structures. Deep learning (Soltanian-Zadeh et al., 2019; Huang et al., 2020; Melinscak et al., 2015) or machine learning algorithms (Turior et al., 2013; Gala et al., 2014; Kandaswamy et al., 2013a; Januszewski et al., 2018) have been proposed to circumvent the requirement for complete neurite segmentation; however, this approach still depends on both inference and manual curation based on a subjective criterion, or complete segmentation of a training data set.

### 1.3. Evaluation of neurite tortuosity using discrete Fourier transforms

We report a solution to the above methodological issues that allows for the inference of neurite tortuosity without the requirement for complete neurite reconstruction or model training. Our solution takes advantage of the fact that by defining tortuosity as a lack of directional coherence of neurites both globally (across the entire image) and locally (grid-wise analysis of subregions within the image), the average tortuosity of a neurite network can be accurately inferred even from a set of discontinuously segmented neurites. Specifically, the method we report measures the degree of anisotropy in the power spectrum of the discrete Fourier transform (DFT) to measure the degree of directional coherence, which we show is inversely proportional to the average tortuosity of the neurites.

## 2. Materials and methods

### 2.1. Animal care

All procedures involving animals were conducted under a protocol approved by the Animal Care and Use Committee, University of California Berkeley, an AAALAC accredited institution. C57BL/6 mice were used. Mice were female and ~ 6 weeks of age. For use in experiments, mice were euthanized using 5% isoflurane (10 min) followed by cervical dislocation and eyes prepared for imaging. Eleven mice were used in this study.

### 2.2. Corneal nerve staining

Enucleated eyes were fixed for 1 h in 100% methanol, followed by washing in PBS for 10 min with gentle rotation. Corneas were then removed under a dissecting microscope while the eye was kept over ice. Dissected corneas were washed once in PBS for 10 min, immersed in blocking solution (3% bovine serum albumin (BSA) and 0.3% Triton X-100 in PBS) for 1 h at room temperature, then incubated for 1 h in 20 mM EDTA at 37 °C. Corneas were then incubated in a primary antibody labeling solution consisting of rabbit anti-mouse β-Tubulin III (Sigma-Aldrich; #T2200) diluted 1:500 in blocking solution and incubated overnight at 4 °C. Corneas were then removed, washed in PBS for 10 min, then incubated with secondary antibody labeling solution consisting of goat anti-rabbit antibody (Life Technologies; #A11034) diluted 1:500 in PBS with 1 × DAPI. After 2 h at room temperature, corneas were transferred to fresh PBS and washed 3 times for 10 min. Each of the above washes and incubation steps involved gentle rotation. Corneas were then flat-mounted with Prolong Gold (Thermo Fisher Scientific, Molecular Probes #P363961) to enhance the visibility during imaging.

### 2.3. Image acquisition

Flat-mounted corneas were imaged using an upright Olympus Fluoview FV1000 Confocal Microscope equipped with a 20x/1.0NA water-dipping objective under. Eyes were imaged using the 488 nm laser line of an argon laser. Z-stacks were imaged over a 636 μm × 636 μm × 74 μm volume with 1.24 μm × 1.24 μm × 1.24 μm cubic voxels. Image stacks were collected from five fields per sample including one from the center of the cornea and four from the peripheral corneal flat petals.

### 2.4. Image processing and analysis

Initial image processing and analysis was performed using FIJI (Schindelin et al., 2015) (ImageJ 1.52p) with the TransformJ (Meijering et al., 2001) plugin and Canny Edge (Canny, 1986) plugin. Statistical analysis and plotting were performed with Python 3.8.3 64-bit with Pandas 1.1.3 (McKinney, 2010) and Microsoft Excel 16.0 64-bit. ImageJ macros and Python code available here: https://github.com/Llamero/Dendrite_tortuosity_analysis.

Statistical analysis was performed using the Kolmogorov-Smirnov (KS) test in SciPy 1.5.2 (Virtanen et al., 2020).

## 3. Results

### 3.1. Image stack segmentation and skeletonization

**Fig. 1.**
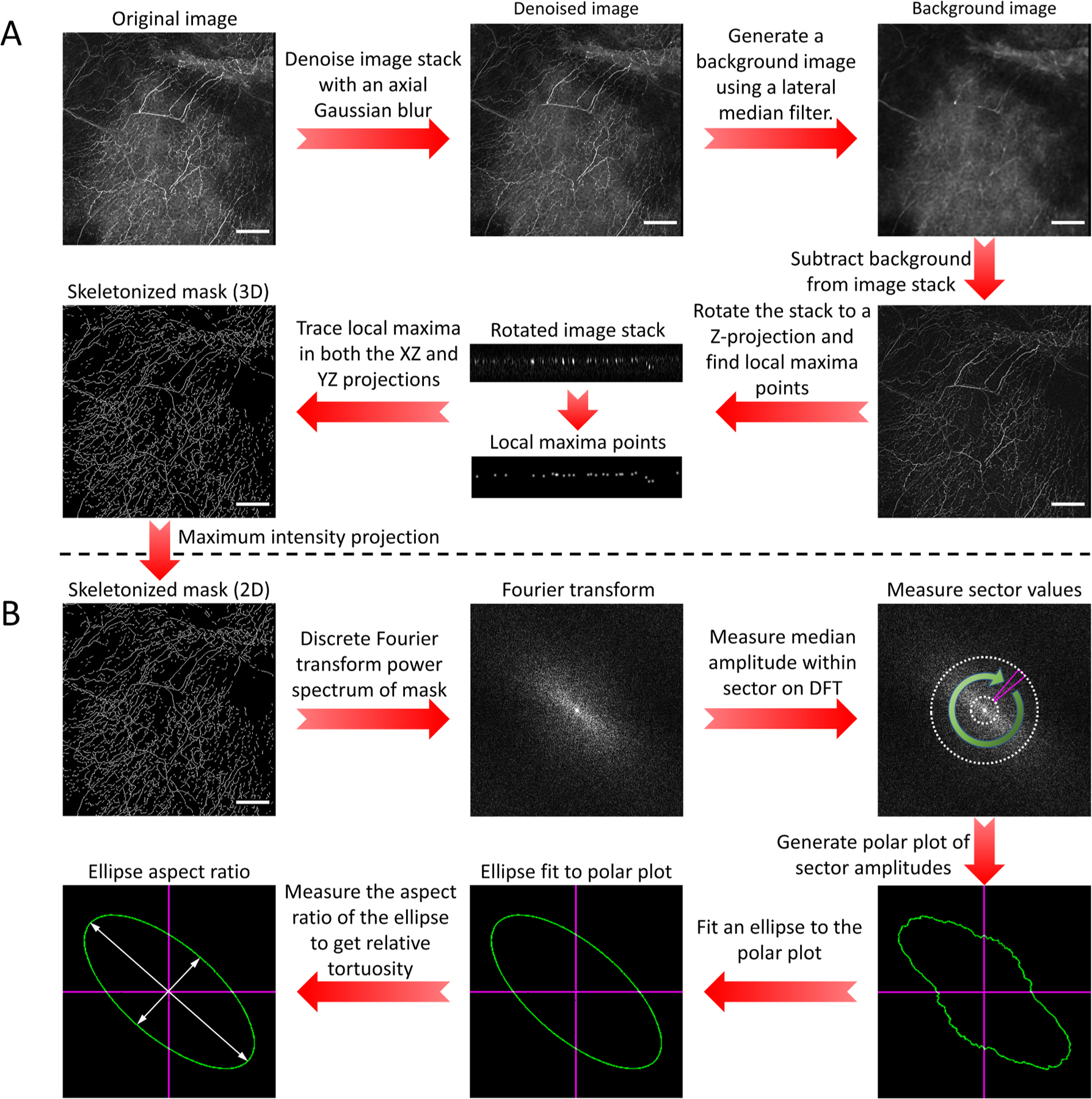
Neurite tortuosity analysis pipeline. (A) Image processing steps used to generate a mask of neurites from a raw image stack. Scale bar = 100 μm. (B) Image analysis steps used to quantify neurite tortuosity using the discrete Fourier transform (DFT) power spectrum. Scale bar = 100 μm.

Prior to analyzing neurite tortuosity, we first segmented and skeletonized the neurites (Fig. 1A). This step was essential to normalize the image stacks and prevent the tortuosity analysis from being biased towards nerve bundles and brighter/thicker neurites. Specifically, we used only standard denoising filters and a simple segmentation/skeletonization approach. The output of this filtering is a two-dimensional binarized mask of all visible neurite segments within the field-of-view. As our analysis method does not depend on the neurite segmentation being continuous, the processing pipeline did not require model fitting or manual curation techniques to bridge the neurite segments, which served to reduce the complexity and time required for initial image processing.

#### 3.1.1. Noise and background subtraction

The high temporal frequency photomultiplier noise was first removed from the image stack using an axial Gaussian convolution. While conventionally a lateral median filter is used to remove detector noise, this filter would also have removed many of the finer neurites in the image, biasing the analysis towards thicker neurites. However, as the Z-step distance between optical sections was smaller than the axial optical resolution, a small axial Gaussian filter (σ = two pixels) suppressed the high frequency detector noise without impacting the axial resolution of the neurites (Fig. S1).

The local contrast and intensity of the neurites was then normalized by removing any background signal, including artefacts such as vignetting as well as structured background such as autofluorescence. Fortunately, neurites are thin, sparse projections within an image, which easily distinguishes them from the mottled background signal. Therefore, a lateral median filter (radius = three pixels) was used to excise the sparse, thin neurites, while retaining as much background detail as possible. The background signal was then be removed by subtracting the median filtered background image stack from the denoised image stack.

#### 3.1.2. Skeletonizing neurites

After the noise and background had been removed from the image stack, the neurites were segmented. Skeletonizing the neurites not only served to segment them into a binary mask, but also normalized their intensity and thickness. This normalization was necessary to prevent thicker neurites and nerve bundles from becoming over-represented in the DFT. One standard approach to segment the neurites would be to threshold the image stack to create a binary mask, and then plot the medial axis (i.e. morphological skeleton) of the thresholded mask. The major limitation to this approach is finding a threshold that maximizes neurite segmentation while minimizing the inclusion of any remaining background signals, especially when automating the analysis pipeline. Therefore, we used an alternate approach using local maxima, which effectively thresholds based on local contrast rather than a global cutoff. Specifically, the image stack was transformed 90° about the X and Y axes to the corresponding Z projections and then the local maxima points were plotted for each slice, taking advantage of the fact that corneal neurites are primarily oriented in the lateral plane. Segmentation of both XZ and YZ projections is necessary to ensure that regions of neurites that are perfectly aligned with either axis are completely segmented (Fig. 1A).

### 3.2. Quantifying neurite tortuosity via DFT

Previous methods for quantifying neurite tortuosity relied on having continuous correctly segmented neurites, and then comparing the length of the neurite to the linear distance the neurite traverses. As previously discussed, this adds complexity to the analysis as it requires complete and correct segmentation of neurites, which can be prohibitive in tissues with dense neurite meshes. Recent approaches used to segment dense, discontinuous neurite meshes have used modeling and inference (Quan et al., 2015; Pani et al., 2014; Li et al., 2019). However, while useful for determining neural connectivity, the resulting neurite paths are a combination of the actual neurite paths derived from the original image stack, and the model’s generated paths used to bridge gaps between neurite segments where there was no signal. This presents a new issue of how to design a model to bridge gaps in neurites that does not impose a priori assumptions on the path those neurites would have taken, which would inherently impact the quantification of the tortuosity of that path. Therefore, a method that can infer neurite tortuosity without needing to fill in these gaps would be ideal, as the measurement would be derived purely from the original data.

Our method circumvents the issue of modeling neurite paths by measuring the directional coherence of the neurites to infer the over-all tortuosity of the neurite paths. This inference takes advantage of the fact that neurites that have a no tortuosity (i.e., travel in the shortest path possible) will inherently be directionally coherent along their path - as the only way to have no tortuosity is for the neurite to always be heading in the same direction.

Specifically, we used the power spectrum of the DFT of the neurite masks to quantify directional coherence (Fig. 1B). Since the power spectrum plots the angle and amplitude of all possible spatial frequencies comprising the image, images with a high degree of directional coherence will correspondingly show an enrichment of spatial frequency amplitudes orthogonal to the neurite paths, where the degree of this anisotropy in the power spectrum is inversely proportional to the degree of tortuosity (see Supplemental Movie 1).

#### 3.2.1. Create polar plot of power spectrum

To measure the anisotropy of the DFT, a 2D plot of the DFT power spectrum was produced of the neurite mask. A polar plot of the power spectrum amplitude was then generated by measuring the median amplitude within an annular sector of the power spectrum (Fig. 1B). The use of an annular sector allowed the measurement to be further refined by creating a bandpass filter that enriched for the spatial frequencies of interest (Fig. 1B - white dashed circles). Specifically, the use of a bandpass filter allows for the analysis to be targeted to a specific range of tortuosity feature sizes and serves to suppress pixel harmonic artefacts found in the highest spatial frequencies (Fig. S2). We used a passband of 5.0 μm per cycle to 40 μm per cycle to analyze the neurite masks, and the equivalent pixel frequency passband of 4.0 pixels per cycle to 32 pixels per cycle to analyze the simulated datasets. The median amplitude within the sector was then measured across a full 360° rotation around the power spectrum and plotted as a polar plot of median amplitude vs. angular direction.

#### 3.2.2. Quantifying polar plot anisotropy

Once the 2D power spectrum plot was reduced to a polar plot, the directional anisotropy can then be inferred by fitting an ellipse to the polar plot and measuring the aspect ratio (minor axis radius: major axis radius) of the polar plot.

### 3.3. Scale variant tortuosity

As others have previously shown, neurite tortuosity is also scale variant, meaning that the degree or tortuosity can vary depending on the length scale with which it is measured (Guimarães et al., 2016). One of the easiest ways to conceptualize scale variant tortuosity is with neurites that radiate from an origin, such as with neurites entering the lamina cribrosa in the retina, or neurites spiraling about an origin, such as in the center of the cornea. In the case of the spiraling neurites, tortuosity at large scales, hereinto referred as global, would appear tortuous as the neurites are continuously changing direction across the field of view. However, at the small scale, hereinto referred as local, the radius of the curvature is sufficiently large that the same neurites can appear to be directionally coherent, assuming there is no additional directional variance in the neurites other than the spiral path.

#### 3.3.1. Scale variant tortuosity simulations

**Fig. 2.**
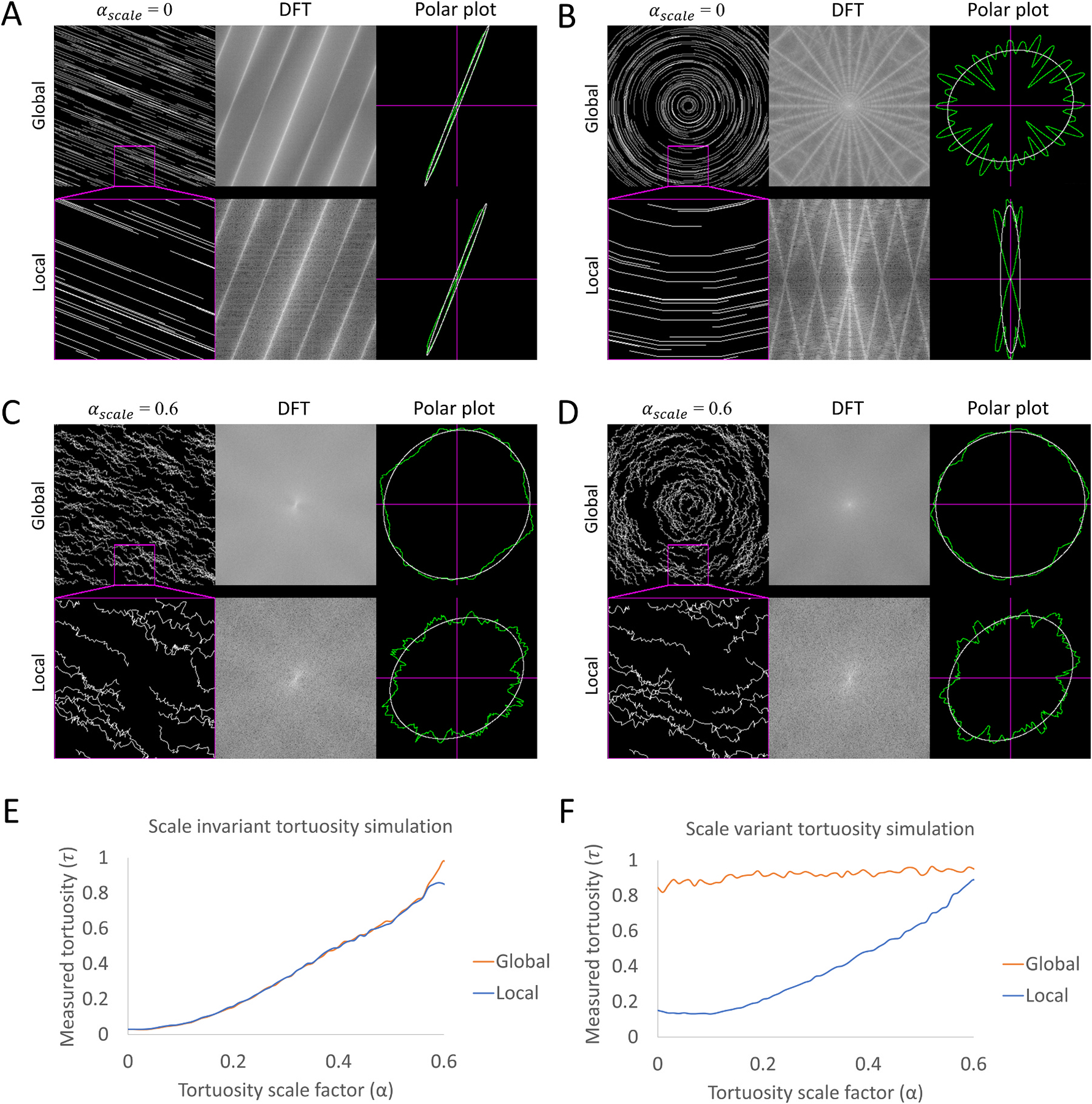
Analysis of stimulated neurite tortuosity. (A) Scale invariant tortuosity simulation where the tortuosity scale factor $\left(\alpha_{scale}\right)=0$. The simulated data was analyzed both with a 1 × 1 grid (global) and 4 × 4 grid (local – magenta inset). The corresponding discrete Fourier transforms (DFTs) and polar plots are also shown. (B) Scale variant tortuosity simulation where the tortuosity scale factor $\left(\alpha_{scale}\right)=0$. The simulated data was analyzed identically to panel A. (C) Scale invariant tortuosity simulation where the tortuosity scale factor $\left(\alpha_{scale}\right)=0.6$. The simulated data was analyzed identically to panel A. (D) Scale variant tortuosity simulation where the tortuosity scale factor $\left(\alpha_{scale}\right)=0.6$. The simulated data was analyzed identically to panel A. (E) Plot of the correlation between the tortuosity scale factor $\left(\alpha_{scale}\right)$ and the measured tortuosity $\left(\tau_{net}\right)$ for both the 1 × 1 grid analysis (global) and 4 × 4 grid analysis (local) of the scale invariant tortuosity simulation. (F) Similar plot to panel E of the scale variant simulation.

To simulate the impact of scale variance when measuring tortuosity, we created both a scale invariant model (Fig. 2A, Supplemental Movie 1), using parallel lines, and a scale variant model (Fig. 2B, Supplemental Movie 2) using lines curving about a central origin. To simulate the discontinuous segmentation of neurites, the number of line segments comprising each line were randomly varied. An identical random seed was used for both simulations, such that the only difference between the two simulations is the starting radius of curvature of each line.

Approximate scale invariant tortuosity was then incrementally added to the simulation by varying the angle between each line segment (Fig. 2C and D) using the following equation: $\theta_{final}=\theta_{initial}+\alpha_{scale}\varphi_{random}$, where $\alpha_{scale}\in \left[0,1\right]$ is the tortuosity scaling factor and $\varphi_{random}$ is a uniform random angle on $\left[-\pi ,\pi \right]$.

#### 3.3.2. Quantifying scale variant tortuosity

The tortuosity quantification method demonstrated in Fig. 1 can be readily adapted to different length scales by dividing the original image into a grid of sub regions, where the dimensions of each grid square represents the length scale in which tortuosity will be measured. One additional step to the method in Fig. 1 is that at smaller length scales, the density of neurites can vary greatly between subregions of an image, so the measured tortuosity of each grid division needs to be normalized to the density of neurites within the grid division using the following equation:

(1) $$\tau_{net}=\frac{\sum_{i=0}^{n}\sum_{j=0}^{n}\tau_{i,j}\rho_{i,j}}{\sum_{i=0}^{n}\sum_{j=0}^{n}\rho_{i,j}}$$

where the original image is divided into an n×n grid, $\tau_{i,j}$ is the measured tortuosity within a grid division, and $\rho_{i,j}$ is the measured neurite density within a grid division (i.e., the fractional surface area of the neurite mask within the grid square).

To validate this quantification method, scale variant and invariant tortuosity was simulated from $\alpha_{scale}=0$ (no tortuosity) to $\alpha_{scale}=0.6$ (high tortuosity). The tortuosity was then quantified globally using a 1 × 1 grid and locally using a 4 × 4 grid (Fig. 2A–D). In the scale invariant simulation, the local and global measured tortuosity were nearly identical and scaled proportionally with the tortuosity scaling factor (Fig. 2E). Conversely, in the scale variant simulation, the global measured tortuosity stayed relatively constant, independent of the scaling factor, while the local measured tortuosity was proportional to the tortuosity scaling factor (Fig. 2F). These results confirm that our method can accurately quantify local and global tortuosity and distinguish between scale variant and scale invariant data and emphasizes the importance of factoring scale when defining and quantifying tortuosity.

### 3.4. In vivo quantification of neurite tortuosity

**Fig. 3.**
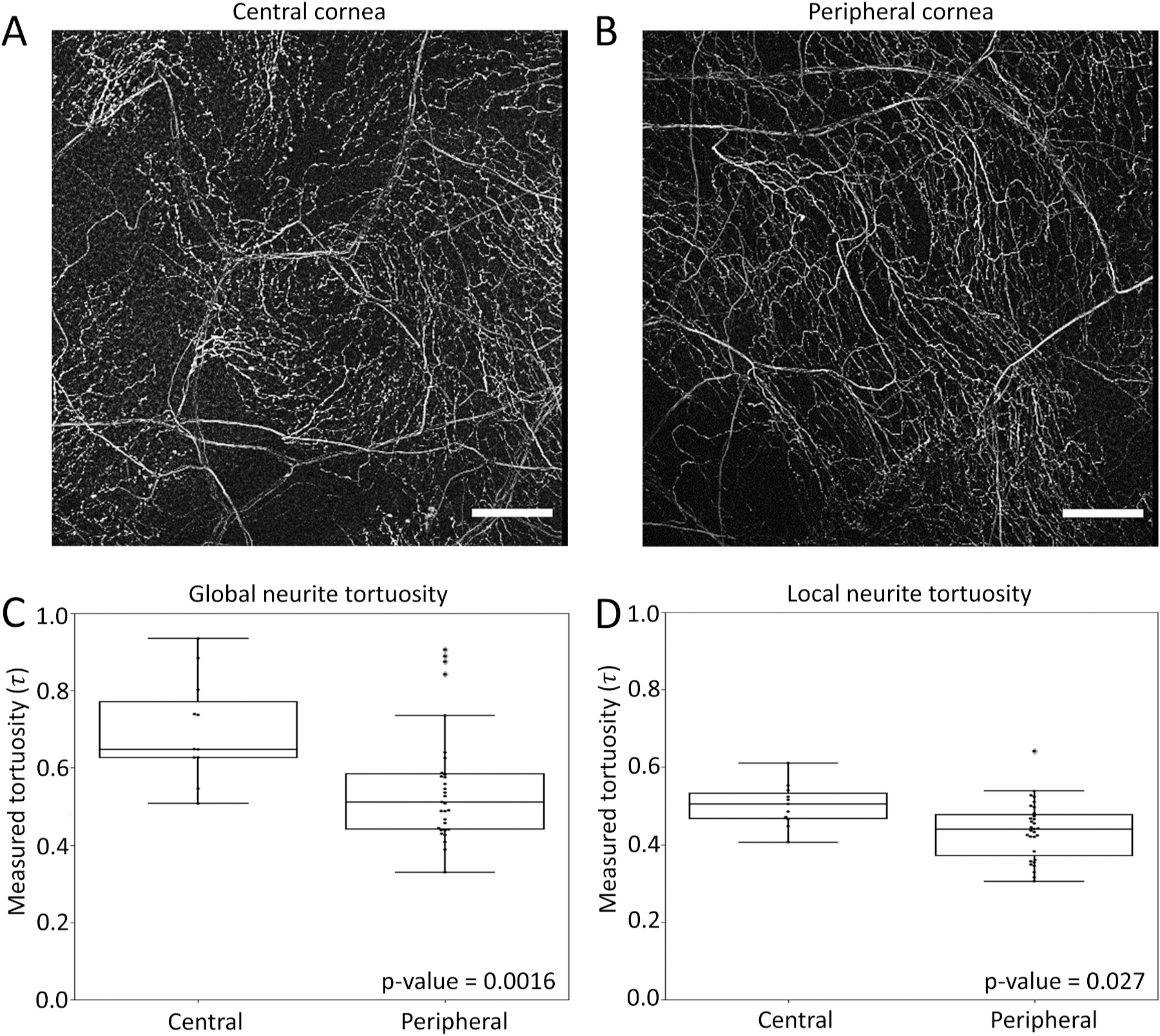
Analysis of corneal neurite tortuosity. (A) Filtered maximum intensity projection of neurites in the murine central cornea. Scale bar = 100 μm. (B) Filtered maximum intensity projection of neurites in the murine peripheral cornea. Scale bar = 100 μm. (C) Notched box plots and swarm plots of 1 × 1 tortuosity grid analysis (global) of central corneal neurite image stacks (n = 11) and peripheral corneal neurite image stacks (n = 31). The respective KS test p-values are shown in the bottom right corner of the plot. (D) Similar plot to C of 4 × 4 tortuosity grid analysis (local).

To validate our analysis method in vivo, we quantified the tortuosity of the neurites innervating the mouse cornea. We specifically chose this model as it is known that the neurites spiral in towards the center of the cornea (Marfurt et al., 2019), resulting in a spiral pattern of neurites at the center of the cornea similar to the scale variant tortuosity simulation (Fig. 3A). Conversely, at the periphery of the cornea, the radius of curvature of the neurite spiral is much larger, causing the neurites to have a pattern similar to the scale invariant tortuosity simulation (Fig. 3B). As a result, we would expect the global scale tortuosity to be different between the central and peripheral neurites, while the local scale tortuosity should be more similar since it would be less impacted by the spiral pattern.

To test this hypothesis, we skeletonized confocal image stacks of neurites from both the central cornea (11 images from 11 mice) and peripheral cornea (31 images from 11 mice) using the method described in Fig. 1A. The neurite tortuosity was then quantified globally using a 1 × 1 grid and locally using a 4 × 4 grid.

At the global scale, the median tortuosity of the neurites in the central cornea was 24% higher than the neurites in the peripheral cornea (delta median = 0.14, KS test p-value = 0.0016) (Fig. 3C). Conversely, at the local scale, there was only an 8.5% difference in neurite tortuosity between the central and peripheral cornea (delta median = 0.064, KS test p-value = 0.027) (Fig. 3D). The fact that the difference in tortuosity between the central and peripheral neurites is three times greater at the global scale than the local scale reveals that the primary difference between the innervation of the central and peripheral cornea is the radius of curvature of the inward spiral which is primarily a global scale effect.

## 4. Discussion

We described a novel approach that allows for measuring the tortuosity of dense, discontinuous neurite networks in vivo without the need for inference or modeling. The outputs of our method therefore reflect only the endogenous tortuosity of the raw data, without possible confounding effects resulting from various computational models that might be used to bridge such discontinuities. We also show that our approach can be tuned to effectively analyze directional coherence not only across a range of distances (global vs. local), but also across specific bands of spatial frequencies (high frequency of inflection points vs. low frequency of inflection points). Since our method was designed for dense, discontinuous neurites common to in vivo conditions, we also suggest it could prove clinically useful for a variety tissues, not just the eye.

### 4.1. Circumventing the problem of discontinuities in existing approaches

A variety of solutions have been proposed to tackle the non-trivial, and at times intractable, issue of accurately and completely segmenting dense and/or discontinuous neurite networks (Quan et al., 2015; Pani et al., 2014; Li et al., 2019). In particular, tracing algorithms are often limited by discontinuities (breaks) in images of neurite networks and have traditionally required some form of model based gap filling approach (Soltanian-Zadeh et al., 2019; Huang et al., 2020; Melinscak et al., 2015) to mitigate this issue. However, as these algorithms require a priori assumptions about the range of valid paths to take when bridging a gap in an image of a neurite, they currently cannot preserve the tortuosity of neurite paths across these discontinuities accurately, as this would require first training the model with the measured tortuosity of the existing data. In other words, accurately measuring tortuosity using existing methods requires bridging neurite discontinuities in the image; however, one needs to know the tortuosity of the neurites to know how to best trace the neurite across the gap, creating a circular argument. The method shown in this paper can be used to resolve this issue by allowing the tortuosity to be analyzed solely based on the raw data available, which could then be used to inform models used to bridge discontinuities in neurites images.

### 4.2. Dense neurite networks

Even in the absence of discontinuities, the issue of accurate and complete segmentation of complex neurite networks remains non-trivial. Manually curated segmentation of just a single image with hundreds or even thousands of individual fibers, can be prohibitively time consuming. This time-cost is further compounded by the fact that experiments often require the segmentation of large image sets, multiplying the labor invested during this process. Many automated approaches continue to be developed to autonomously segment neurites and/or quantify tortuosity (Joshi et al., 2008; Wilson et al., 2008; Bullitt et al., 2004; Koprowski et al., 2012; Scarpa et al., 2011; Grisan et al., 2008; Barch et al., 2021; Heneghan et al., 2002; Kandaswamy et al., 2013b; Jiang et al., 2021; Banerjee et al., 2020); however, accurate segmentation of neural networks remains an open challenge, where current methods still require both manually segmented training sets and manual validation of the results. Our method resolves this issue by quantifying neurites at the population level negating the need to segment individual neurites, allowing for broader application to image sets with dense innervation.

### 4.3. Limitations

Although our method addresses several key issues not accounted for by previously discussed approaches, it is not by any means a universal solution to all neurite types. Our method is best applied to very dense and/or discontinuous neurite images and as it is designed to measure tortuosity at the population level, other methods would be much better suited for inferring the tortuosity of individual neurites in a network. For example, if the image of interest contains singular, well-defined neurites, as seen in 2D neuronal cell cultures (Kandaswamy et al., 2013a), then previously described methods would serve better as they would be able to provide individual neurite tortuosity values which could then be averaged to obtain a population level description. Conversely, our approach should prove sufficient if not desirable in clinical settings as it directly returns values of over-all tortuosity at the population level, the descriptor used in diagnosis of disease pathologies (Edwards et al., 2014; Hamrah et al., 2013; Kurbanyan et al., 2012; Tuominen et al., 2003).

As previously mentioned, this method is also limited to directionally coherent neurites since it infers tortuosity from the degree of directional coherence within the neurites, as measured by the degree of anisotropy in the power spectrum of the DFT. As a result, this method would not be well suited for analyzing radially symmetric/isotropic neurite networks, such as those often found in culture in the absence of a scaffold, or radially symmetric neurons such as amacrine cells (Famiglietti, 1983).

### 4.4. Summary

Overall, our new approach for inferring the average tortuosity of dense and/or discontinuous neurite networks commonly found in images of in vivo innervation, allows scientists and clinicians to bypass the need for accurate complete neurite segmentation or model training. Additionally, the proposed method involves use of standard image processing to skeletonize neurites, removing background signal and artifacts through a series of simple convolutional filters. As a result, this method serves to complement existing methods for measuring tortuosity, expanding the array of datasets of neurite networks that can be easily and accurately quantified.

## Supplementary Material

mmc1

mmc1_lrg

mmc2

mmc2_lrg

mmc3

mmc4

## Figures and Tables

**Movie 1. Supplemental Movie 1.**
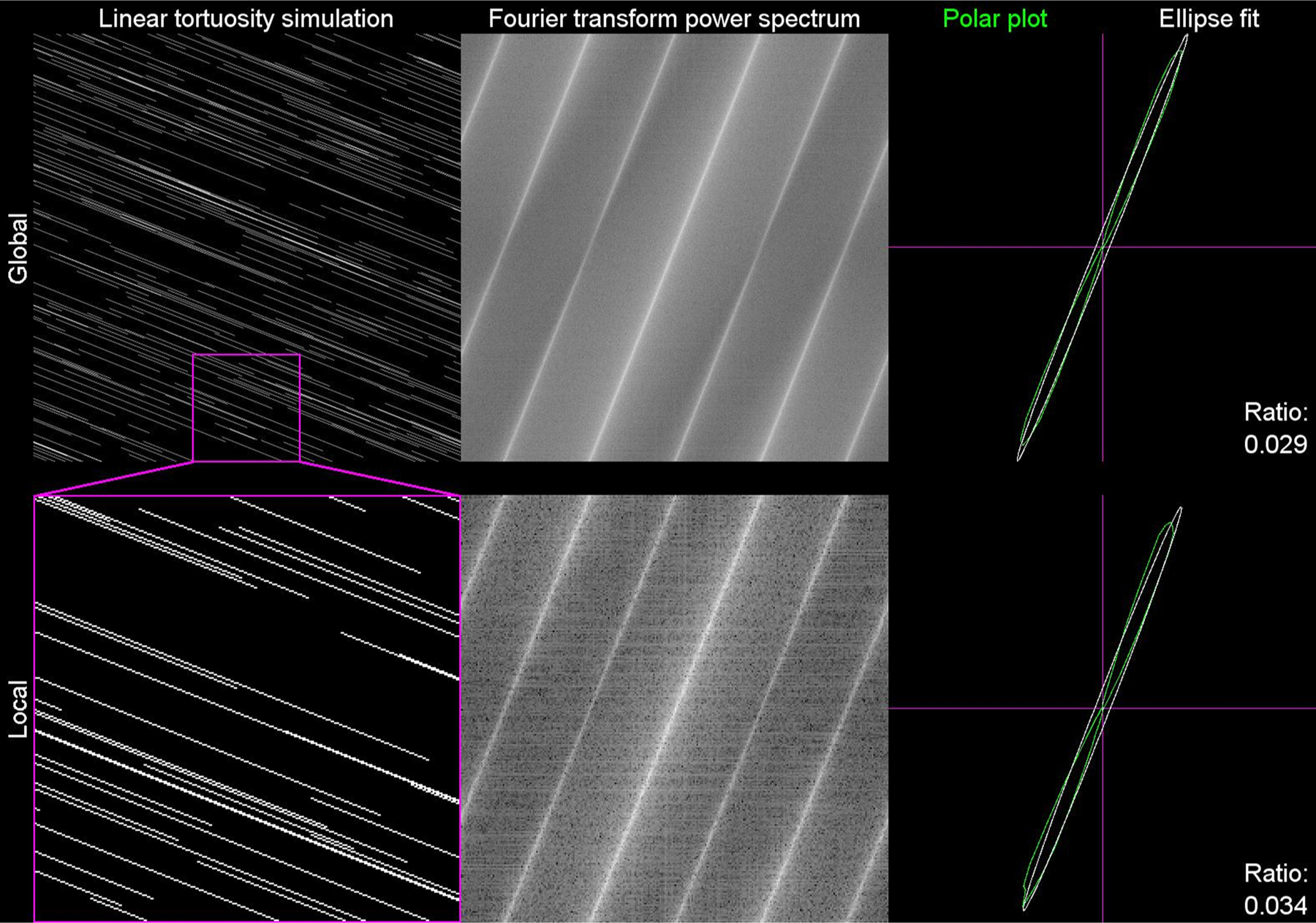
Scale invariant tortuosity simulation and analysis. This movie shows the scale invariant tortuosity simulation with the tortuosity scale factor $\left(\alpha_{scale}\right)$ increasing linearly from 0 to 0.6 (left column). The corresponding discrete Fourier transform (middle column), and polar plot analysis (right column) are also shown. The top row shows the analysis for the whole field, while the bottom row represents a local analysis that is 1/16th the area of the whole field.A video clip is available online. Supplementary material related to this article can be found online at doi:10.1016/j.jneumeth.2021.109266.

**Movie 2. Supplemental Movie 2.**
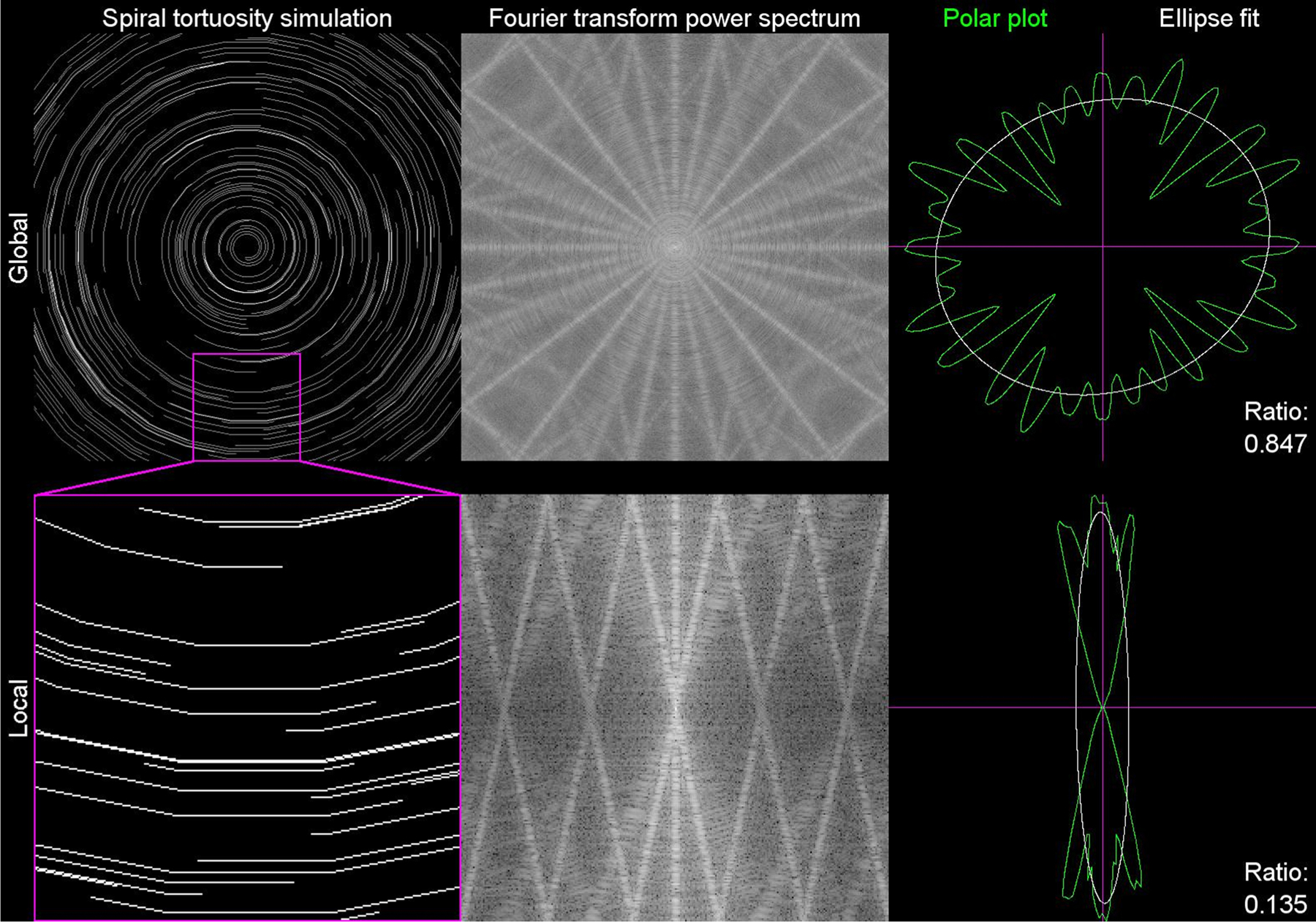
Scale variant tortuosity simulation and analysis. This movie is the same as Supplemental movie 1, but for the scale variant tortuosity simulation.A video clip is available online. Supplementary material related to this article can be found online at doi:10.1016/j.jneumeth.2021.109266.

## Abbreviations:

IVCM: *In vivo* confocal microscopy
ATEF: automatic tortuosity estimation framework
DFT: discrete Fourier transform
KS: Kolmogorov–Smirnov
