# Supplementary figures and images for: Quantification of relative neurite tortuosity using Fourier transforms
Source: J Neurosci Methods. Author manuscript; Available in PMC 2024 Mar 26. (PMC10964090; doi:10.1016/j.jneumeth.2021.109266)

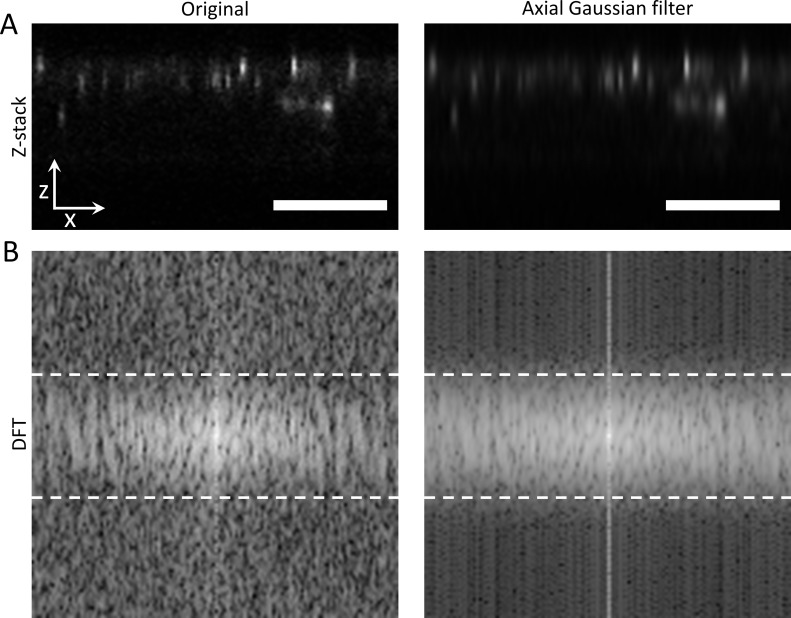

Supplement: mmc1 [file NIHMS1972141-supplement-mmc1.jpg]

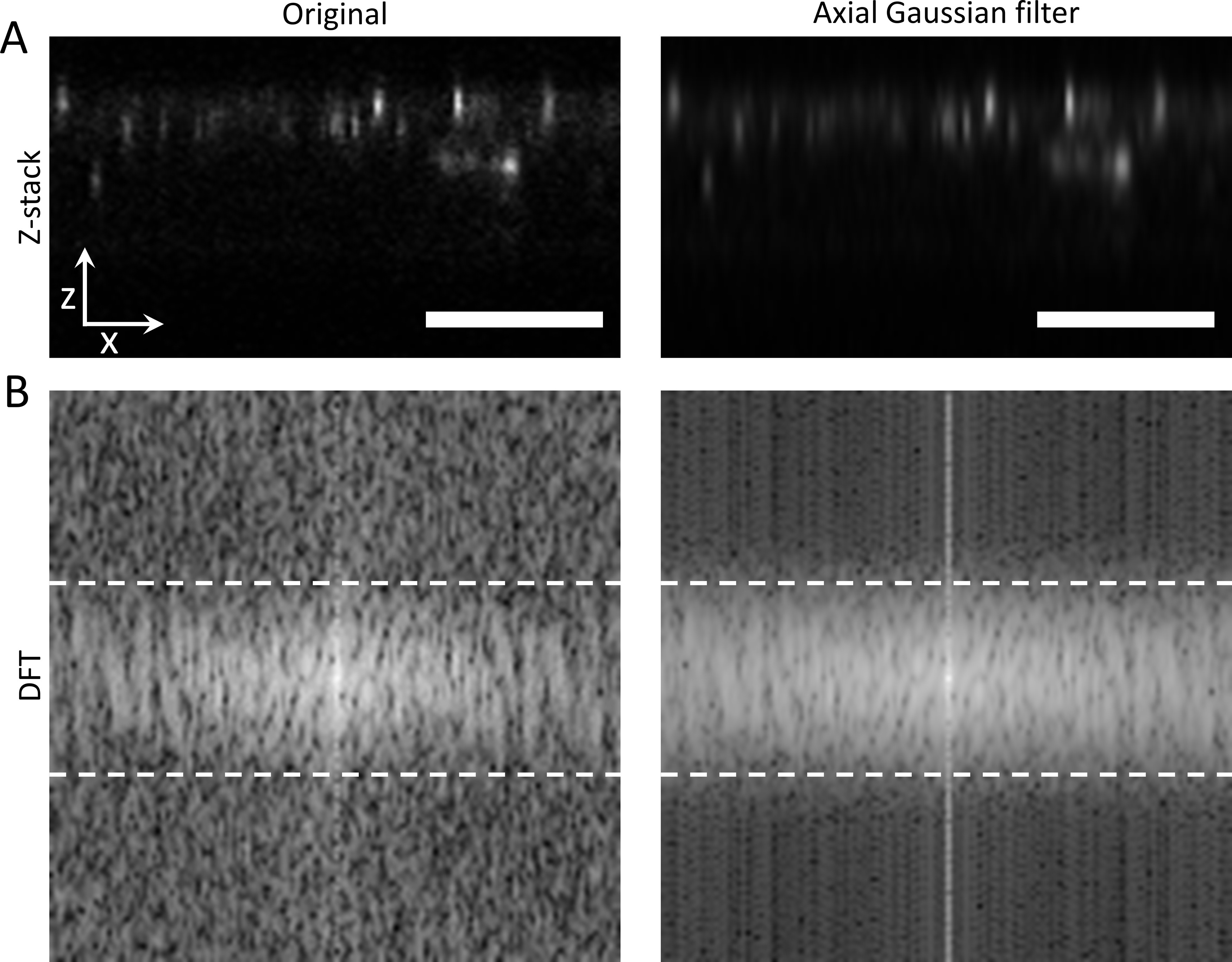

Supplement: mmc1_lrg [file NIHMS1972141-supplement-mmc1_lrg.jpg]

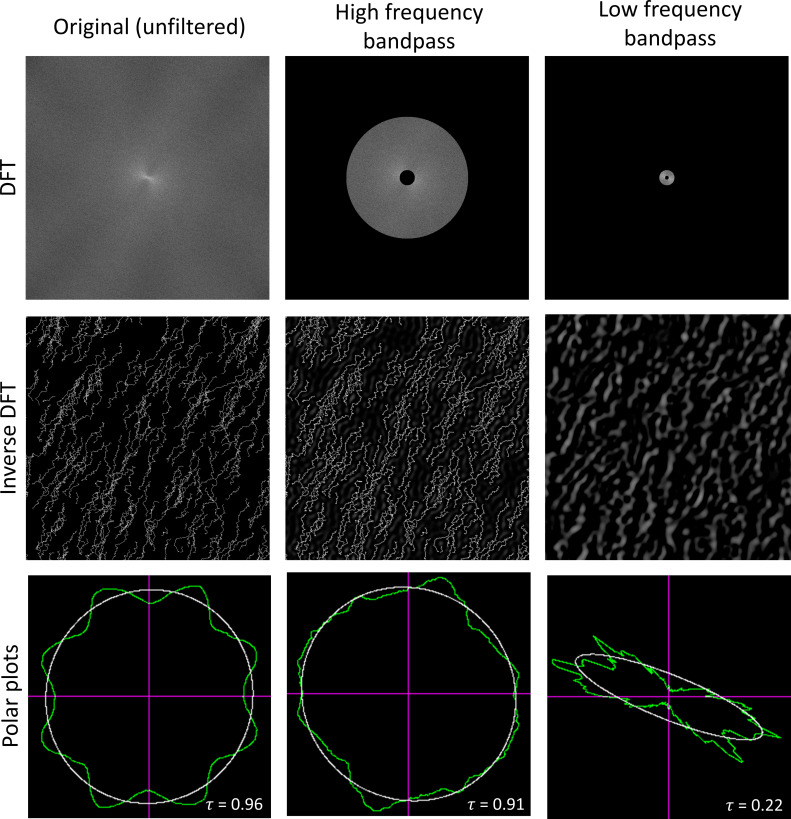

Supplement: mmc2 [file NIHMS1972141-supplement-mmc2.jpg]

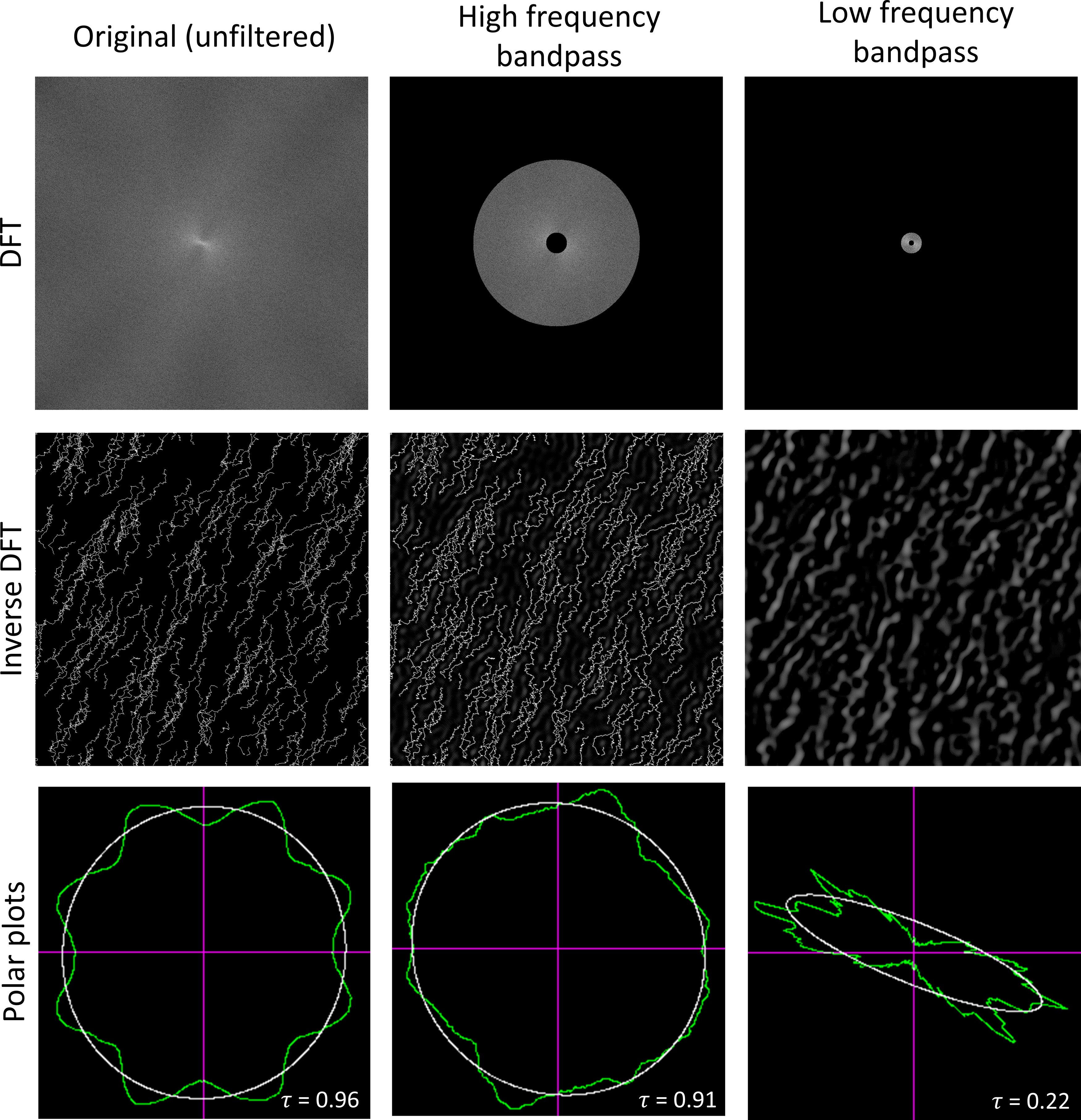

Supplement: mmc2_lrg [file NIHMS1972141-supplement-mmc2_lrg.jpg]
